# Twilight Anæsthesia in Labour

**Published:** 1916-09-16

**Authors:** F. Barlow


*To the Editor of* The Hospital.

### Twilight Anæsthesia in Labour.

*To the Editor of* The Hospital.

Sir,—Twilight sleep, that condition of somnolent indifference to pain during childbirth induced by the administration of Hyoscine and Opium derivatives, has of late been widely extolled in the lay Press. Extravagant claims have been made for those satisfactory points about this procedure which patently appeal to the lay mind; of these the most impelling is the absence of pain, whereas important details which materially tincture the advisability of universal application have been ignored, suppressed, or misunderstood. The whole theme, as was to be expected, has been ravenously seized upon by women desirous of avoiding pain at all cost, who, seeing this twilight sleep exploited by journals and periodicals on all sides, have quite naturally ill-digested the principle and failed to appreciate the many valid objections there are to its general adoption. The lay writers, who understand but little the dangers to which the patient is exposed, except in selected cases, realise still less the exactions imposed on the doctor by this method unless conducted under special surroundings. They would have every parturient woman semi-comatose, regardless to every other consideration except that of abolishing her consciousness to pain.

It is true that great improvements have recently been made in the drugs used to procure twilight anæsthesia (*e.g.*, the tendency of scopolamine to decompose has been effectually countered by the addition of mannite), and, owing to this, the state of the child is now relatively satisfactory. All those who ask, or rather demand, from the general practitioner that every confinement be conducted under twilight anæsthesia should be made to realise that the great difficulty, the insuperable obstacle which prevents its widespread adoption, is the fact that it is essentially an institutional method which is quite unsuitable for private practice. This point will be well borne in upon them when they are made to understand, that the doctor must be in *constant* attendance during the *whole* progress of labour. He must for the time being have only one patient, must relinquish every other call of his practice and concentrate his whole attention, time, and energy on the patient who desires twilight anæsthesia. From this it is quite obvious that the ordinary scale of confinement fees can have no application here, nor can they even remotely be taken as a guide.

The unequivocal attention and unremitting presence demanded of the medical attendant by twilight anæsthesia will be appreciated by considering the following facts :

The only suitable cases in which twilight anæsthesia is safe are those where a normal, uncomplicated delivery is anticipated as suggested by previous examination of the patient. The mother must be normal and the child in a normal presentation. It is essential to examine the patient at frequent intervals, since it is only thus that the exact progress can be gauged. A normal case following a normal course would require approximately the following treatment : As soon as the pains due to genuine uterine contractions have definitely set in with persistent recurrence, and have attained a frequency of roughly one every five minutes or so, the first injection is given (intramuscularly in the gluteal region). This consists of Hyoscine Hydrobromide (Scopolamine) gr. $\frac{1}{150}$ coupled with Omnopon gr. $\frac{1}{3}$, or Narcophine gr. $\frac{1}{2}$, or Morphia gr. $\frac{1}{3}$. About one hour later the second injection is given, consisting of Scopolamine gr. $\frac{1}{150}$. [Some prefer to divide this dose into two, thus injecting Scopolamine gr. $\frac{1}{300}$ one hour after the first injection, and half an hour after this another injection of Scopolamine gr. $\frac{1}{300}$ if necessary.]

About two hours after the first injection the third injection is given, consisting of Scopolamine gr. $\frac{1}{300}$ in conjunction with Omnopon gr. $\frac{1}{6}$, or Morphia gr. $\frac{1}{6}$, or Narcophine. The interval between the second and third and subsequent injections is regulated by the condition of the patient, the depth of twilight anæsthesia being the criterion of frequency. After the third, further injections will be required at approximately hourly intervals. These usually consist of Scopolamine gr. $\frac{1}{300}$ alone without Omnopon, Morphia, or Narcophine, though these can be added if thought necessary. When delivery can be expected to occur in from one to two hours further injections are suspended lest the child should be adversely affected.

The ideal condition is one in which the patient is somnolently conscious between the pains, slightly restless and annoyed when the pains rouse her to a sense of discomfort, but with absolute loss of memory—to coin a word, she is in a nepenthupnotic condition (*νηπένθη*=forgetfulness, *ὕπνος*=sleep).

Care must be taken not to give too large a dose at any time nor to inject too frequently, lest uterine contractions become weak or cease altogether.

It is difficult to estimate the average number of injections required, since it varies with the essential and adventitious circumstances and with the individual under treatment. As a general rule, subject, however, to wide variation, about half a dozen injections will be necessary for primiparæ. But cases are on record in which up to twenty injections have been given in twenty-four hours, without any harmful results to mother or child being observed. Occasionally the patient passes into a state of unmanageable excitement towards the end of labour, in which case an inhalant anæsthetic must be given. The room in which twilight anæsthesia is to be conducted must be absolutely quiet and have the light shaded.

The advantages of twilight anæsthesia are stated to be that it is especially adapted to highly-strung women, and particularly so in neurotic primiparæ, but equally indicated in the case of multiparæ, since it substitutes in place of the apprehension, and even terror, of the ordeal of childbirth, an utter calm disregard or even indifference to the pains inalienably associated with this period. Another advantage is the lessened liability of damage to the perinæum.

The disadvantages and dangers that suggest themselves are the prolongation of labour, which is stated to take about one-third longer by this method than normally; the liability to respiratory affections of the new-born child which is marked, since in the majority of cases the child is partly asphyxiated when born. In view of this the fœtal heart-sounds should be frequently and carefully noted throughout parturition, and any alteration taken as an intimation to discontinue twilight anæsthesia and to adopt prompt and effective measures to deliver without delay. Another danger is the probability of the symptoms becoming masked in cases that are merging into the abnormal unless the patient is very frequently examined. If any manipulations become necessary or the application of forceps essential, an anæsthetic will have to be given, since the patient has no self-restraint and cannot be controlled. Occasionally persistent restlessness and continuous thirst are a troublesome feature throughout.

The after-effects so often noticed with twilight anæsthesia consist of severe headache lasting for days, dizziness, unquenchable thirst, a general feeling of malaise, restlessness going on in some cases even to delirium.

Provided the medical attendant can give his undivided attention, is constantly with the patient, that the confinement is conducted in an institution with trained nurses to look after the patient, that she is under continuous and close observation, that the case is uncomplicated by any abnormality either in mother or child, twilight anæsthesis can be looked upon as a relatively safe procedure.—Yours faithfully,

F. BARLOW, L.R.C.S.E., L.R.C.P.E., L.R.F.P.S.G.

